# Using Abattoir-Based Surveillance to Establish Foot-and-Mouth Disease Non-Structural Protein Seropositivity in Cattle and Pigs in Cambodia

**DOI:** 10.3390/ani15111624

**Published:** 2025-05-31

**Authors:** Lida Kong, Jarunee Siengsanan-Lamont, Sothyra Tum, Paul W. Selleck, Jeeranan Areerob, James R. Young, Laurence J. Gleeson, Stuart D. Blacksell

**Affiliations:** 1 Mahidol-Oxford Tropical Medicine Research Unit, Faculty of Tropical Medicine, Mahidol University, Bangkok 10400, Thailand; lida@tropmedres.ac (L.K.); hjjar@yahoo.com (J.S.-L.); paul.selleck@csiro.au (P.W.S.); thisisnan2225@gmail.com (J.A.); laurence.j.gleeson@gmail.com (L.J.G.); 2 National Animal Health and Production Research Institute, General Directorate of Animal Health and Production, Khan Mean Chey, Phnom Penh 120603, Cambodia; sothyratum@gmail.com; 3 School of Veterinary Science, The University of Sydney, Camden, NSW 2570, Australia; cowvet@gmail.com; 4 Nuffield Department of Medicine, Centre for Tropical Medicine & Global Health, University of Oxford, Oxford OX3 7LF, UK

**Keywords:** livestock, FMD, swine, seroprevalence, abattoir, transboundary animal disease, Southeast Asia

## Abstract

Foot-and-mouth disease (FMD) is a contagious transboundary animal disease that causes economic loss and obstacles to international trade. Frequent FMD outbreaks in Cambodia negatively impact farmers’ and smallholders’ incomes. This study aimed to estimate the seroprevalence of FMD Non-Structural Protein (NSP) antibodies, which are an indicator of FMD antibodies raised during a natural infection rather than those produced following vaccination, that were detected using a commercial enzyme-linked immunosorbent assay (ELISA). Sample collection from cattle and pigs (*n* = 2238) was performed at ten abattoirs in seven provinces between October 2019 and December 2020. Overall seroprevalence in cattle and pigs was 43.2% (363/839; 95% CI 39.8–46.7), and 0.6% (9/1399; 95% CI 0.2–1.2), respectively. Only the cattle dataset was included in the risk factor analysis, as the prevalence of sero-reactors was too low in the pig dataset to be analyzed. Significant risk factors identified by the logistic regression model included the province of origin (*p* = 0.02), body condition score (BCS) (*p* = 0.0002) and sex (*p* = 0.0007). Odds ratios of the significant risk factors were 7.05 (95% CI 1.43–34.67; *p* = 0.02) for cattle that originated from Kampong Thom, 1.41 (95% CI 1.05–1.89; *p* = 0.02) for female cattle, and 3.28 (95% CI 1.06–10.12; *p* = 0.04) for animals with BCS of 3/5. The study revealed that the seroprevalence of FMD NSP in cattle presented at the abattoirs was high, while the FMD NSP seroprevalence in abattoir pigs was very low. Further investigation is required to map the disease distribution in Cambodia, especially the serotypes and strains causing clinical disease. These findings call for the extension of work on effective disease prevention measures.

## Simple Summary

Foot-and-mouth disease (FMD) is a serious disease affecting cloven-hoofed animals that spreads easily and causes financial losses, especially for farmers in Cambodia. This study examined the presence of FMD antibodies in cattle and pigs to understand infection levels. Researchers tested 2238 animals at ten slaughterhouses in seven provinces between October 2019 and December 2020. Results showed that 43.2% of cattle had FMD Non-Structural Protein (NSP) antibodies, while only 0.6% of pigs tested positive. Because so few pigs were affected, further analysis focused only on cattle. The study found that certain factors were significantly associated with the presence of antibodies against the non-structural proteins of the FMD virus: cattle from Kampong Thom province, female cattle, and those with a moderate body condition score (BCS 3/5) had a higher risk. These findings demonstrated a potential method for surveillance of FMD NSP antibody monitoring at the abattoir, providing a surveillance tool to be used to assess the success of FMD control. The results indicated FMD is widespread in Cambodian cattle, while pigs are less affected. More research is needed to track the disease’s spread and identify specific virus types. The study highlights the need for better prevention strategies to protect livestock and farmers’ livelihoods.

## 1. Introduction

Foot-and-mouth disease (FMD) is a viral disease considered one of the most contagious animal diseases and affects particularly cloven-hoofed animals [1]. The virus can be transmitted through different pathways, such as direct contact between infected and susceptible animals or indirect contact via contaminated objects, e.g., shoes, clothes, vehicles, and other fomites [2]. It is a serious animal health problem in many developing countries, including Cambodia [3], as the virus causes high morbidity, which reduces livestock productivity, including milk output and draught capacity, and causes weight loss [4]. However, it generally has a low mortality rate, except in very young animals, where mortality can be significant [5]. Cattle and pigs are both important FMD-susceptible livestock species, and the movement of livestock plays a crucial role in FMD dissemination [6,7]. FMD virus (FMDV) is endemic in Southeast Asia, with a recorded number of 4961 FMD outbreaks within the period of 2007–2017 [8]. Serotypes O, A, and Asia1 have been reported in Cambodia [8,9]. However, animal disease surveillance was not regularly performed in the past, and there is still limited information on the FMD situation in Cambodia [10], suggesting novel surveillance methods are needed.

According to the Cambodian Agriculture Sector Strategic Development Plan for 2019–2023, livestock production was considered a priority sub-sector in the agricultural sector and plays an essential role in improving smallholder income and generating the resultant socio-economic benefits [11]. However, most Cambodian livestock producers face severe constraints due to limited marketing access, climate change, and other endemic diseases [12]. Cambodia is also a member of the South-East Asia and China Foot-and-Mouth Disease (SEACFMD) campaign coordinated by the World Organization for Animal Health (WOAH). The General Directorate of Animal Health and Production (GDAHP) annual report indicated that FMD outbreaks occur annually in Cambodia [13]. Control measures to stop or reduce the spread of the FMD include movement restrictions, improved biosecurity, and vaccination [14]. As supplies and resources for more widespread and preventive administration are limited, vaccines for FMD control in Cambodia have mainly been used in ring vaccination around outbreaks or provided by donors during specific aid and research projects. To this end, the government has supported these limited FMD vaccination activities conducted by the Department of Animal Health and Veterinary Public Health (within the GDAHP) in collaboration with the Provincial Animal Health and Production Offices [13]. Historically, a range of vaccines have been used and have included Afropop^®^ (Merial) Trivalent, Oil Adjuvanted, Inactivated Purified Vaccine against Foot and Mouth Disease (virus strains: O Manisa, O-3039, A May 97, Asia-1, Merial), Aftopor^®^ (Merial) Monovalent, Oil Adjuvant, Inactivated Purified Vaccine against Foot and Mouth Disease (virus strains: O Manisa, O 3039), and Raksha-Ovac (Indian Immunologicals Limited) Trivalent, Oil Adjuvant, Killed Vaccine against Foot and Mouth Disease (Virus strains: O, A and Asia-1) at the recommended dose [15]. The control approach of differentiating infected from vaccinated animals (DIVA) is generally applied in situations where FMD is not endemic, and emergency vaccination is used to control an outbreak. In the case of Cambodia, where disease is endemic, multiple serotypes have been in circulation, vaccination is somewhat sporadic, and animal history at the point of sampling is somewhat vague, the NSP ELISA is an ideal tool to get an indication of the level of FMDV infection in the cattle population. One drawback of the approach is that if animals have been vaccinated multiple times or with inadequately purified vaccines, then NSP antibody will be induced, leading to incorrect assignment of positive results.

An earlier survey study of smallholder farmers who participated in an ACIAR-funded project to assess their knowledge of biosecurity and disease reported that only 28.3% of all farmers correctly identified purchasing unvaccinated cattle as a risk for disease transmission, and only 15.4% of farmers correctly stated that selling sick cattle is a risk for disease transmission [16]. When asked what disease causes cattle to have tongue, teat, and foot ulcers, 44.2% correctly identified FMD [16]. In a survey of smallholder cattle farmers in 2015 from four provinces, 32.1% reported their cattle had been vaccinated for FMD within the last 12 months [16]. It should be noted that in this project, FMD vaccines were offered to smallholder farmers at no cost, and therefore, results do not reflect the broader adoption of FMD vaccination [16].

Financial modeling investigating the net benefit of vaccinating cattle for FMD has demonstrated positive outcomes at both the individual and national levels [4]. A partial budget analysis identified a strong positive incentive for cattle to be vaccinated biannually for FMD, providing an estimated benefit of USD 31.48 per animal for each animal owned [4]. A benefit-cost analysis (BCA) of a vaccine-led FMD control program over a 5-year period, using ad hoc modeling to estimate the true FMD incidence, indicated that an annual vaccine program where all large ruminants were vaccinated twice (at a cost of USD 3.15 per vaccine per animal) would cost approximately USD 26 million per year and had the potential to avoid losses of USD 135 million if a large-scale outbreak occurred in the first year of the program, leading to a benefit-cost ratio of 6.05 [17].

The current survey study of animals presenting for slaughter aimed to investigate the seroprevalence of FMDV non-structural protein (NSP) antibodies that are present in animals that have been infected, but generally not in animals that have been vaccinated. This approach was considered the most efficient way to detect FMDV infections in a population where three different serotypes are known to circulate, where vaccines are sporadically used, and where there are limited resources to conduct more structured surveillance. The outputs of this abattoir surveillance system provided baseline information on FMD prevalence, which will contribute to Cambodia’s control and prevention strategies, as well as demonstrating a novel method for FMD surveillance in a resource-limited environment.

## 2. Materials and Methods

### 2.1. Study Design

**Figure 1:**
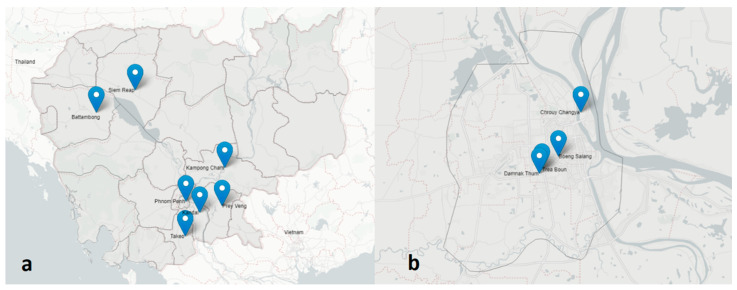
Study abattoir location map: (**a**) Cambodia; (**b**) Phnom Penh Province.

Ten abattoirs were included in the cross-sectional study from seven provinces, namely Phnom Penh (4 abattoirs), Battambang, Takeo, Siem Reap, Prey Veng, Kandal, and Kampong Cham (Figure 1a). The sampling was performed between October 2019 and December 2020. The sample size calculation was based on Cannon and Roe’s technique using an expected prevalence of approximately 10% and test sensitivity of 80% and above [18]. Each sample collection round aimed to collect a minimum of 30% of the animals slaughtered on the sampling day. Abattoirs included in the study were purposively selected to target seven provinces in the country’s central region based on high livestock movement and also accessibility, particularly through the rainy season. During the 4-month trial period, from October 2019 to January 2020, sample collections were performed in four abattoirs in Phnom Penh (Boeng Salang abattoir processing both cattle and pigs, Chhrouy Changya abattoir processing cattle, and Damnak Thum and Trea Boun abattoirs processing pigs only, Figure 1b). The study was then implemented in the other six provinces from June to December 2020, with abattoir selection based on the high throughput of animals slaughtered per night. In Phnom Penh, the samples were collected only from the Boeng Salang abattoir between September and December 2020.

### 2.2. Sampling and Sample Collection

During the first trial period in Phnom Penh, the sampling team planned to visit each abattoir (Boeng Salang, Chhrouy Changya, Damnak Thum, and Trea Boun abattoirs) monthly. The Boeng Salang abattoir, which processed both cattle and pigs, was planned to be visited twice monthly. However, the total visits to these abattoirs varied from three to six trips. For the later phase, the plan was to visit an abattoir in each of the seven provinces, including Phnom Penh, monthly (a total of 7 visits to each). However, due to COVID-19 restrictions, the survey in Phnom Penh was not resumed until September 2021, which reduced the number of planned visits to 3. Provincial abattoir veterinary officers collected blood samples and animal information under the supervision of the National Animal Health and Production Research Institute (NAHPRI) staff as part of on-the-job training exercises. A blood sample was collected aseptically from swine and cattle’s ear or jugular vein using single-use disposable syringes and then transferred to an anticoagulant-free vacutainer and individually labeled. The tubes were placed in a rack, sealed in a large zip-lock plastic bag and kept in a cool box with ice packs while being transported immediately by field staff to the NAHPRI laboratory in Phnom Penh. If an animal was unable to be sampled safely, the next available animal was selected. Animal information, including the type of animal sampled, body condition score (BCS) (1 = extremely thin, 2 = thin, 3 = moderate, 4 = fat, and 5 = extremely fat), age (or estimated based on teeth wear if not known), sex, the origin (province) of the animal, breed, and vaccination status, was also collected where the information was available.

### 2.3. Sample Handling and Processing

The serum was extracted from each blood sample by spinning in a refrigerated centrifuge (Thermo Scientific, Germany) at 1000–2000 rpm for 10 min. The serum samples were stored at 2–8 °C while testing and kept at −20 °C or lower for long-term storage.

### 2.4. FMD NSP Serological Analysis

All serum samples were tested for antibodies against the FMD NSP using an enzyme-linked immunosorbent assay (ELISA) commercial kit, ID Screen^®^ FMD 3ABC NSP competition ELISA (Cat# FMDNSPC-5P0; ID Vet, Grabels, France). The procedure for detecting antibodies in serum was based on the manufacturer’s instructions. The result was read with an ELISA microplate reader (Infinite F50, Singapore), and then an S/N% was calculated using the ID softTM software version 5.05 [19]. Based on the manufacturer’s procedure, S/N%, less than or equal to 50%, was considered a positive result (S/N% ≤ 50% = positive), and greater than 50% was considered negative (S/N% >= negative).

### 2.5. Data Analysis

The ELISA test results were summarized and entered into Microsoft Excel (2020). Statistical analyses were performed in R studio version 4.1.0 [20]. Descriptive statistics were applied, including the percentage, frequency, and proportion of the seropositive and seronegative samples. The Fisher’s exact test (for the low prevalence dataset) or Chi-square test was used to determine associations between the dependent variable (test result) and the independent variables (country of origin, abattoir province, province of origin, BCS, sex, and age). As cattle age was deemed only an estimation, animals were grouped into ≤3 and >3 years of age. Significant variables with a *p*-value ≤ 0.1 were then included in the multivariate logistic regression models through a stepwise process. Independent variables with a variance inflation factor (VIF) equal to or higher than ten were omitted from the model to avoid multicollinearity [21]. The final model was selected using the Akaike Information Criterion (AIC) and the Hosmer and Lemeshow test for the goodness of fit. Odds ratios of significant independent variables (*p*-value < 0.05) were also calculated. Maps showing the animal’s province of origin and destination provinces were created using the R-studio leaflet package [22] for visualization.

## 3. Results

### 3.1. Seroprevalence of FMDV NSP Antibodies in Cattle

**Figure 2:**
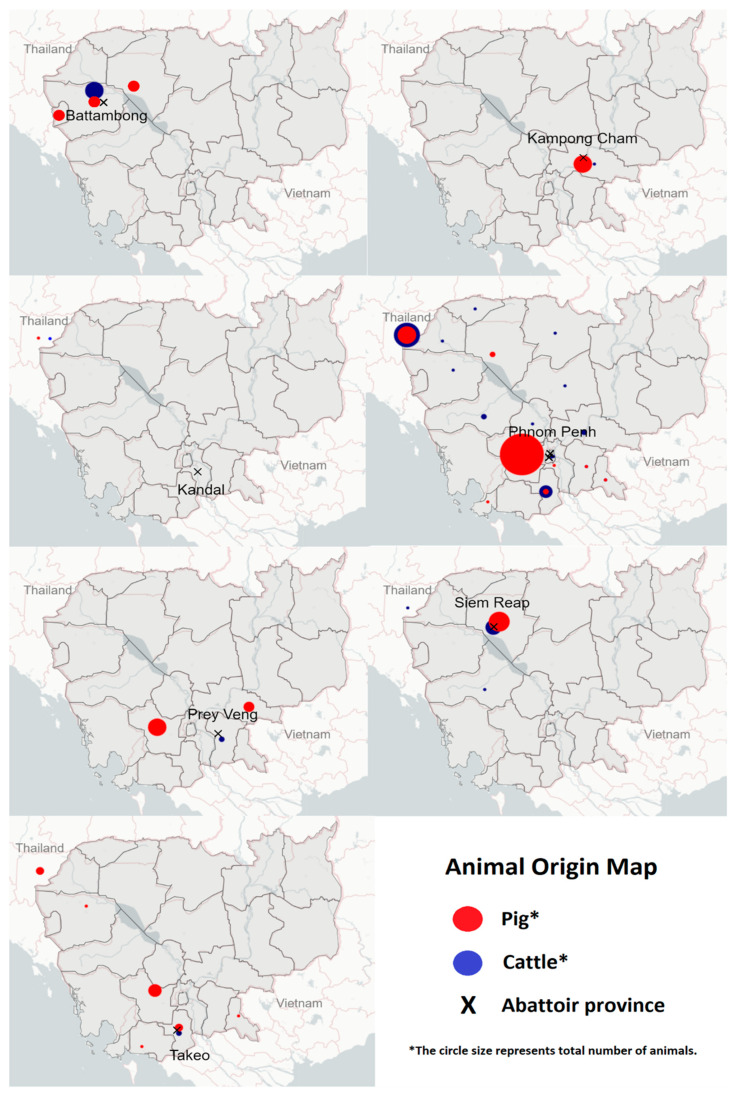
Map of Cambodia indicating animal origin for each of the seven provincial abattoirs sampled. From the top left and then clockwise: Battambang, Kampong Cham, Phnom Penh, Siem Reap, Takeo, Prey Veng, and Kandal.

A total of 2238 serum samples were collected from pigs (*n* = 1399) and cattle (*n* = 839) (Table 1). A summary of seroprevalence by the province of origins is presented in Table 2, while the movement of animals is shown in Figure 2. Data on vaccination status and animal breed were not available. No buffalo samples were collected in this study, as they did not present at any of the abattoirs where sampling occurred. Sample collection in Kandal and Kampong Cham provinces was only performed on one occasion due to a deficit in human resources.

**Figure 3:**
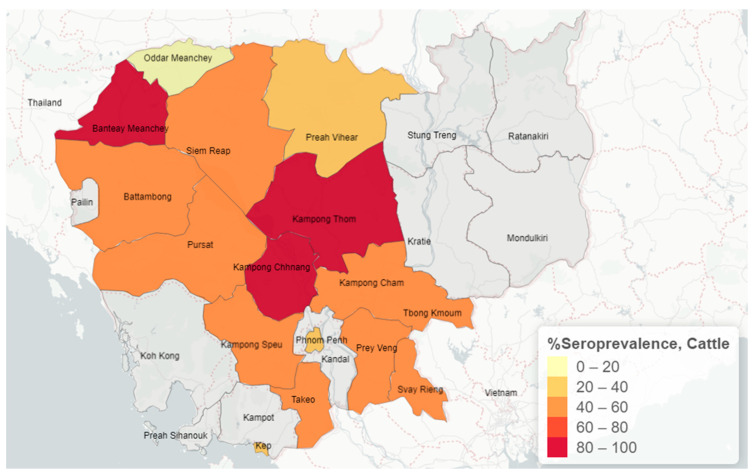
Map of FMD non-specific protein seroprevalence for cattle based on province of origin.

The overall FMD NSP seroprevalence results were 43.2% (363/839, 95% CI 39.8–46.7) in cattle. As per abattoir locations, the seroprevalences of FMD NSP antibodies were 54.5% (60/110) for Battambang, 52.3% (57/109) for Siem Reap, 45.1% (14/31) for Prey Veng, 40.2% (220/547) for Phnom Penh, and 37.5% (12/32) for Takeo (Table 1). For cattle, seroprevalence per origin province is illustrated in Figure 3. Out of 839 cattle, 362 animals had a BCS noted. The highest seroprevalence was for cattle with BCS 3 (88/152; 57.9%), followed by BCS 2 (25/44; 56.8%) and BCS 4 (75/150; 50%), while the lowest prevalence was for cattle with BCS 5 (5/16; 31.3%). No cattle were recorded with a BSC of 1. Concerning animal origin, the prevalence of FMD NSP antibodies in the local cattle was 47.4% (286/603), while the prevalence of cattle imported from Thailand was 32.6% (77/236) (Table 3). The proportion of FMD NSP antibodies in male cattle was 38.0% (*n* = 458), while in female cattle it was 49.6% (*n* = 381). Factors associated with FMD NSP seropositivity with a *p*-value ≤ 0.1 identified by univariable analysis included country of origin (*p* < 0.001), abattoir province (*p* < 0.01), province of origin (*p* < 0.01), sex (*p* < 0.001), BCS (*p* < 0.001), and age (*p* = 0.096). These significant variables were included in the multivariate logistic regression analyses. The final model included province of origin (*p*-value = 0.02), BCS (*p* < 0.001), and sex (*p* < 0.001). Odds ratios of the significant risk factors were 7.05 (95% CI 1.43–34.67; *p* = 0.02) for the cattle originating from Kampong Thom province compared to the animals imported from Thailand, 1.41 (95% CI 1.05–1.89, *p* = 0.02) for female cattle over male cattle, and 3.28 (95% CI 1.06–10.12; *p* = 0.04) for cattle with BCS 3 compared to those with BCS 5.

### 3.2. Seroprevalence of FMD NSP in Pigs

The overall seropositive rate of FMD NSP in the sampled pigs was 0.6% (9/1399). Regarding the sampling abattoir location, the seropositive FMD NSP samples were located in two provinces, namely Phnom Penh [abattoirs: Boeng Salang (0.4%; 1/245), Damnak Thum (1.6%; 3/185), Trea Boun (1%; 3/295)], and Takeo (1.0%; 2/191) (Table 1). Most pigs slaughtered originated from either various commercial farms in Cambodia, were imported from Thailand, or were raised by smallholders (Table 3). The seroprevalence of FMD NSP found in commercial farms and imported from Thailand was 0.6% (5/833) and 1.6% (4/250), respectively (Table 2). Only pigs from a single commercial farm in Kampong Speu province and Thailand were seropositive, and the study did not detect seropositive samples in any smallholder pigs (*n* = 306). The age of pigs ranged between 3 and 7 months old, which fits with local ages of pigs sent to market. Most sampled pigs were 4 months (55.5%; *n* = 726), followed by 5 months (44.4%; *n*= 669), 3 months (0%, *n* = 3), and 7 months (0%, *n* = 1). Fisher’s exact test showed no significant correlation between seroprevalence and independent variables.

## 4. Discussion

In Cambodia, while there are influenza [23,24] and Japanese Encephalitis [25,26] studies in pigs and wildlife zoonoses [27] studies, which are collaborations between international organizations and government-funded research institutes, there is a general lack of published scientific information on animal disease surveillance in large ruminants and limited studies conducted on transboundary livestock movements. Besides the seroprevalence and risk analysis outcomes, an important component of this study was the support and on-the-job training provided to the provincial veterinary officers who participated in the surveillance activities. Even though the surveillance locations and information gathered were limited, the outcomes provided a snapshot of the FMD situation in Cambodia. The survey design was planned to collect approximately 30% of the abattoir holding of animals required for slaughter on the sampling day. Thus, the total number of samples collected on each sampling date varied. One major constraint observed by our study was limited staff capacities in sample collection and biosecurity and biosafety techniques when working with live pigs and cattle. While traders were aware of the animal’s province of origin, the collection of the animal’s history proved difficult, as “middlemen” and traders did not know vaccination status or specific breed history.

An earlier serological study, conducted in 2006 in the southern provinces of Cambodia by Tum et al. [9], indicated that the seroprevalence of FMD NSP in cattle was 30.0% (95% CI 27.1–33.0; *n* = 277). The current study, performed 13 years later, would indicate that the FMD situation in cattle has worsened. Recently, news media reported FMD outbreaks in numerous provinces in Cambodia [28,29]. Farmers commonly reported disease information to village animal health workers or private veterinarians and were willing to pay for treatments and vaccinations. According to the World Animal Health Information Database (WAHIS Interface), GDAHP annually reported FMD outbreaks in cattle and buffalo in several provinces of Cambodia. However, no FMD has been reported in pigs since 2020 [13]. Even with an annual vaccination program in place, 928 FMD outbreaks, distributed all over Thailand, have been reported to the ASEAN Regional Animal Health Information System from 2007 to 2017 [8]. In neighboring Lao PDR, previous studies reported that the FMD NSP seroprevalence in pigs was 1.3% (*n* = 597) in 2019 [30] and 8.2% (*n* = 4851) in 1999–2001 [31]. Results from both countries suggest that pigs are not an important reservoir for FMD infection in the livestock population, similar to earlier findings in Thailand [32].

In recent years Cambodia imported large numbers of cattle and pigs from Thailand as the local smallholder supply did not meet the growing demands for meat consumption, particularly during Cambodia’s festive seasons [6,33]. Such importation increases the risk of spreading transboundary animal diseases [34], including classical swine fever [35], recently introduced lumpy skin disease [36], or African swine fever (ASF) [37]. The movement of animals in the country is also difficult to control [34]. More than one-third of pigs and cattle slaughtered in Phnom Penh were imported from Thailand, and the remainder of the pigs were from large commercial farms owned by multinational companies. It should be noted that, as Thailand had not reported ASF at the time of the study, pigs were still legally allowed to be imported [38]. Additionally, it was notable that due to ASF outbreaks in 2019 in Vietnam, no pigs from that country were imported due to the GDAHP imposing a ban on pig importation to prevent the cross-border spread of the disease [39]. GDAHP and the Ministry of Agriculture, Forestry and Fisheries (MAFF) of Cambodia also announced a plan to reduce pig importation to support local pig production, help boost smallholder income, and decrease the illegal transit of live pigs from Thailand to Vietnam through Cambodia [11,40]. Even though Cambodia reduced the number of live pigs imported from Thailand in 2020 [41], some risks of FMD transmission still exist, as our study detected FMP NSP seropositive imported pigs from Thailand.

Regarding cattle, risk factor analysis demonstrated that Cambodian cattle, especially those originating from Kampong Thom province, had higher exposure to FMDV than imported cattle from Thailand. The cattle in Cambodia typically feed on native grassland in paddy fields for several months after the rice harvest and share grazing areas with animals from other locations, increasing the risk of contracting diseases, particularly FMD, as previously reported by other studies [42,43]. Animal movement management was one of the main challenges faced by the government and stakeholders in controlling livestock diseases in the region [6]. A previous study in Cambodia demonstrated that the movement of live animals played a crucial role in disease transmission and may be responsible for disease introduction and its subsequent spread [7,44].

Another risk factor was that female cattle were 1.5 times more likely to have FMD NSP antibodies than male cattle. A previous study demonstrated that farmers kept females longer than males for breeding purposes, and cattle were kept in groups and released to pastures daily [9]. The same study by Tum et al. [9] also revealed that seroprevalence in adult animals was similar to our research. Some studies stated that infection-induced FMD NSP antibodies could persist for a long duration [45,46], potentially persisting for several years [47]. In our study, even though the seroprevalence of the cattle aged more than three years was higher than less than or equal to 3 years group, statistical analysis showed no significant difference between the seroprevalence of these groups. Unlike pigs, the slaughter of cattle is performed at random ages, reflecting the decision to send cattle to slaughter based on smallholders’ need for cash as opposed to fattening cattle for the market.

Our study identified that cattle with lower BCS had significantly higher FMD NSP seroprevalences, similar to the finding previously reported from Bangladesh [48]. Animals with a high BCS probably indicate that animals are on a higher quality feed ration, better husbandry and biosecurity measures are employed, and therefore, there is less chance of infection. Additional protective factors might be an increased likelihood of vaccination in well-raised animals or a lower probability of contacting diseased animals because of other husbandry practices. The lower BCS could also be a result of FMDV infection. As BCS is a subjective measure, the recording of BCS could also be a source of bias.

There is no available data on the number of FMD vaccine doses used annually in Cambodia. It has been reported that vaccines incorporating FMD serotypes O, A, and Asia are used in Cambodia [9]. Regarding FMD vaccine availability, monovalent and trivalent vaccines were used in Cambodia [9], but the overall FMD vaccine coverage was reported to be relatively low [43,49]. For effective control, it is recommended that an estimated 85% of the susceptible population be vaccinated to provide adequate protection and prevent the dissemination of the virus [50,51]. The Government of Cambodia and international agencies have supported FMD vaccination programs; however, vaccines were mainly provided for emergency response purposes to suspected outbreak areas reported by provincial animal health officers and not routinely distributed to all locations due to limited funding support. Efforts should also focus on supporting the establishment of sustainable commercial FMD vaccine suppliers so that they can register, distribute, and sell into the Cambodian market, particularly as financial benefits for vaccination have been demonstrated [4,42]. One study reported that the private pig production sector followed vaccination programs and applied farm biosecurity to prevent FMD outbreaks [9]. Current control measures are not adequate to reduce the endemicity level; therefore, it is likely that the viruses will continue to circulate and limit livestock production potential for the foreseeable future.

This study has several limitations. Firstly, there is a lack of exact information regarding the current FMD vaccination history of the study animals presented at the abattoir as well as vaccination practices and vaccine sources in Cambodia in general. In some of the provinces of origin, there were low numbers of animals tested, which may lead to sampling bias when interpreting provincial prevalence. The use of non-purified FMD vaccines [9] or repeated doses of purified vaccines [52] can produce NSP antibodies that would result in no discrimination between naturally acquired and vaccine-derived antibodies by the NSP ELISA, resulting in false positive test outcomes. Furthermore, the study is restricted to the slaughtered animals at 10 abattoirs in seven provinces within Cambodia; therefore, the results do not represent Cambodia as a whole. Abattoir sampling has disadvantages from the epidemiological perspective in that the sources of the sampled animals are often not known, sampling is not random, and in the case of FMD, neither is vaccination history. There may also be infection bias in the population presented. However, the prevalence of NSP antibodies in this purposive sample population, especially if estimated over a number of sampling intervals, is still a useful indicator of the overall burden of infection and therefore disease.

## 5. Conclusions

The overall seroprevalence of FMD NSP antibodies in abattoir cattle was high, unlike in pigs. The results suggested that FMD is endemic in Cambodia. However, our study could not determine the disease distribution or its origin because this study only targeted slaughtered animals in a limited area. Even though the abattoir-based survey presented some limitations, such an exercise provides much-needed capacity building at the provincial level and helpful information on disease prevalence and potential risk factors, particularly in light of the absence of other routine surveillance. We conclude that abattoir-based surveillance offers a cost-effective and practical option for low-resource countries, and this would be further strengthened with fresh virus sample analysis. The limitations identified in the study could be addressed in future research to strengthen conclusions. The outcomes also highlighted the need to strengthen the country’s animal disease surveillance and reporting system and staff capacity at the provincial level. It is recommended that better monitoring and recording of FMD vaccines used in the field will help support seroprevalence study interpretations. In addition, field reports of FMD should be actively investigated with fresh lesion samples collected for serotyping, which would aid in improving knowledge of circulating FMDV and vaccine selection.

## Figures and Tables

**Table 1:** FMD non-specific protein (NSP) antibody prevalence for each abattoir.

Province/Abattoir Name	Cattle			Pigs		
	Total	Positive	NSP Prevalence % (95% CI)	Total	Positive	NSP Prevalence % (95% CI)
Phnom Penh/Boeng Salang	207	83	40.1 (33.3–47.1)	245	1	0.4 (0.0–2.2)
Chrouy Changya	340	137	40.2 (35.0–45.7)	-		-
Damnak Thum *	-		-	185	3	1.6 (0.3–4.6)
Trea Boun *	-		-	295	3	1.0 (0.2–2.9)
Battambang/Krong Battambang	110	60	54.5 (44.7–64.0)	125	0	0.0 (0.0–2.9)
Takeo/Krong Doun Kaev	32	12	37.5 (21.1–56.3)	191	2	1.0 (0.1–3.7)
Kampong Cham/Krong Kampong Cham	3	0	0.0 (0.0–70.7)	27	0	0.0 (0.0–12.7)
Siem Reap/Krong Siem Reap	109	57	52.2 (42.5–62.0)	127	0	0.0 (0.0–2.8)
Kandal/Krong Ta Khmau	7	0	0.0 (0.0–41.0)	21	0	0.0 (0.0–16.1)
Prey Veng/Neak Loeung	31	14	45.1 (27.3–64.0)	183	0	0.0 (0.0–2.0)
Overall	839	363	43.2 (39.8–46.7)	1399	9	0.6 (0.2–1.2)

* Denotes abattoir that only slaughtered pigs.

**Table 2:** FMD non-specific protein antibody (NSP) prevalence for cattle and pigs and their province of origin.

Province of Animal Origin	Cattle		Pigs	
	Total	NSP Prevalence %	Total	NSP Prevalence %
Battambang	122	50.8 (62/122)	68	0
Bonteay Meanchey	1	100 (1/1)	-	-
Kampong Cham	49	47.0 (23/49)	14	0
Kampong Chhnang	3	100 (3/3)	-	-
Kampong Speu	39	43.6 (17/39)	603	0.8 (5/603)
Kampong Thom	10	80.0 (8/10)	-	-
Kampot	-	-	12	0
Kap	3	33.3 (1/3)	-	-
Kandal	-	-	6	0
Oddor Meanchey	1	0	-	-
Phnom Penh	16	31.2 (5/16)	-	-
Preash Vihear	12	33.3 (4/12)	-	-
Pailin	-	-	33	0
Prey Veng	34	41.1 (14/34)	20	0
Pursat	42	47.6 (20/42)	-	-
Siem Reap	105	53.3 (56/105)	191	0
Sihanoukville	-	-	31	0
Svay Reing	4	50 (2/4)	9	0
Takeo	162	43.2 (70/162)	83	0
Tboung Khmom	-	-	68	0
Thailand	236	32.6 (77/236)	250	1.6 (4/250)
Unknown	-	-	11	0
Total	839	43.2 (363/839)	1399	0.6 (9/1399)

**Table 3:** Characteristics and demographics of FMD non-specific protein antibody seroprevalence in cattle and pigs.

Categories	Total	Positive	Seroprevalence (95% CI)	*p*-Value (Chi-Square)
Cattle				
Import	236	77	32.6 (26.6–39.0)	
Local	603	286	47.4 (43.3–51.5)	0.0001
Male	458	174	38.0 (33.5–42.6)	
Female	381	189	49.6 (44.4–54.7)	0.0009
Less than or equal to 3 years	392	165	42.0 (37.1–47.1)	
More than 3 years	447	198	44.2 (39.6–49.0)	0.566
Pigs				
Import	250	4	1.6 (0.4–4.0)	
Unknown	10	0	0 (0–30.0)	
Commercial	833	5	0.6 (0.1–1.3)	
Smallholder	306	0	0 (0–1.1)	
Female	799	6	0.7 (0.2–1.6)	
Male	600	3	0.5 (0.1–1.4)	

## Data Availability

Data can be made available at the request of the corresponding author.
